# Membrane BioReactor (MBR) Activated Sludge Surrogate Alternatives Carboxymethyl Cellulose and Xanthan Gum: A Statistical Analysis and Review

**DOI:** 10.3390/membranes12100909

**Published:** 2022-09-20

**Authors:** Nicolás Ratkovich, Rafael Amaya-Gómez

**Affiliations:** Chemical Engineering Department, Universidad de los Andes, Cra 1 Este No 19A-40, Bogotá 111711, Colombia

**Keywords:** MBR, activated sludge, carboxyl methyl cellulose, xanthan gum, viscosity

## Abstract

Membrane Bioreactors (MBR) combine traditional biological treatments such as Activated Sludge (AS) with a membrane-based filtration process to extract suspended and organic solids. MBR operation involves high shear rates near the membrane surface due to the high crossflow velocity, which complicates any simulation process from a hydrodynamic point of view. In this regard, the viscosity as a function of total suspended solids (TSS) plays an essential role in characterizing and modeling the behavior of activated sludge (AS). However, AS has an intransparency property that prevents experimental measurements (i.e., velocity profiles) commonly associated with optical techniques from being peformed. In light of this limitation, two polymeric compounds, carboxymethyl cellulose (CMC) and xanthan gum (XG), are considered here in order to explore the possibility of mimicking the rheological behavior of AS. These compounds are commonly used in the food industry as food thickeners, and their rheological behavior is supposedly well defined in the literature. In this work, we reviewed the viscosity behavior of these compounds through their reported flow behavior and consistency indexes. It was found that the rheological properties of these two polymers differ depending on the chemical manufacturer, rheometers, and measurement protocols involved. Different curves (shear rate vs. viscosity/shear stress) are obtained, as each device and procedure seem to modify the polymer structure. Therefore, a statistical analysis was performed based on the flow and consistency indexes using different concentrations and temperatures reported in experimental data. Several insights regarding CMC, XG, and AS performance were obtained, including a better relationship with concentration than with temperature or certain exponential-based performances, which can support further MBR design and operational decision-making.

## 1. Introduction

Membrane bioreactors (MBRs) combine membrane separation technology with biological treatment. The main problem with MBRs is membrane fouling, which results in the need for frequent cleaning and even membrane replacement, considerably increasing operational costs. In terms of operation, three aspects are considered decisive in fouling the membrane: particle size distribution (PSD), biological parameters, and process hydrodynamics. Due to the complexity of the MBR process, the modeling exercise is broken down into the development of several sub-models. For the hydrodynamics process, an essential factor for MBR models is the activated sludge (AS) viscosity, as this parameter determines the hydraulic regime and transport phenomena near the membrane to a large extent. However, as Activated Sludge presents challenges during experimental measurements such as determining velocity profiles because of its intransparency, different compounds are required to mimic the rheological behavior of the AS.

Viscosity (μ) is defined by the ratio between shear stress (τ) and shear rate ($\dot{\gamma }$), as presented in Equation (1):

(1) $$\mu =\frac{\tau }{\dot{\gamma }}$$

**Figure 1:**
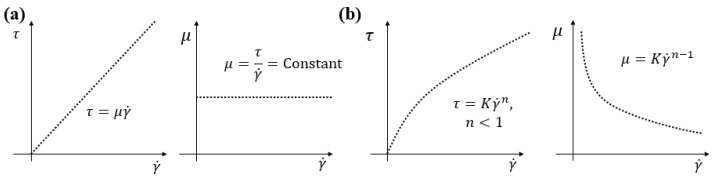
Schematic shear stress vs. shear rate and viscosity vs. shear rate for (**a**) Newtonian and (**b**) pseudo-plastic fluids.

The viscosity of Newtonian liquids (e.g., water) exhibits a linear shear stress–shear rate relationship and constant viscosity. However, particulate suspensions such as AS, as well as polymeric compounds such as carboxymethyl cellulose (CMC) and xanthan gum (XG), exhibit pseudo-plastic non-Newtonian behavior [1,2,3]. In this case, the shear stress is related to the shear rate according to a power-law relationship, as presented in Equation (2):

(2) $$\tau =k\left(C,T\right){\dot{\gamma }}^{n(C,T)}$$

where *n* is the flow behavior index, which has a value less than one for pseudo-plastic (shear-thinning) fluids, and K is the flow consistency index ($Pa\cdot s^{n}$). Both of them depend on the temperature (*T*) [4] and concentration (*C*). Combining Equations (1) and (2), the viscosity is defined by Equation (3), obtaining the behavior shown in Figure 1.

(3) $$\mu =k\left(C,T\right){\dot{\gamma }}^{n(C,T)-1}$$

A temperature-dependent relation for the flow consistency index is provided by an exponential Arrhenius type relation [5], as presented in Equation (4).

(4) $$k=k_{C}\exp\left(\frac{E}{R_{gas}T}\right)$$

where *E* is the activation energy, $R_{gas}$ is the universal gas constant (8.3145 J·K^−1^·mol^−1^), and *T* is the temperature in Kelvin. The flow behavior index is presented in Equation (5), where *a* is constant.

(5) $$n=N_{C}\exp\left(a\;T\right)$$

This work aims to review the main findings from these rheological properties of carboxymethyl cellulose (CMC) and xanthan gum (XG). These compounds and the activated sludge (AS) are tentatively well-described in the literature. In this regard, 37 references dealing with different rheological parameters of CMC were found, 13 references for XG, and 17 for AS, all of which were analyzed in detail. The rest of this paper is structured as follows: Section 2 describes applications from the literature of the non-Newtonian liquids CMC and XG in terms of reactors, two-phase flow in pipes, bubble columns, electrodiffusion methods, and rheological measurements. It also reviews the main findings on activated sludge for Membrane Bioreactors. Section 3 compares the main rheological parameters of CMC, XG, and AS and presents statistical trends from a regression and classification point of view. Finally, Section 4 presents concluding remarks.

## 2. Activated Sludge for Membrane Bioreactos and Applications of Non-Newtonian Liquids (CMC and XG) in the Literature

Rheological characterization is considered an appropriate tool to understand the behavior of wastewater sludge suspensions and improve the operation of wastewater treatment processes. However, to proceed to a correct rheological characterization of fluids such as WWT sludge it is necessary to pay close attention to the choice of relevant measuring geometries and experimental procedures.

Brannock et al. [6] developed a CFD model of two full-scale MBRs. Based on experimental measurements, the viscosity of AS was a minimum of five times higher than that of water. For that reason, a power-law model was incorporated into the CFD model. Comparison with the tracer test showed that the AS rheology had minimal effect. Further investigation of the hydrodynamics showed that this could be attributed to the high turbulent viscosity ratio present within the system.

Defrance et al. [7], Xia et al. [8], and Zhao et al. [9] developed specific correlations of AS viscosity for the operating range of their systems. Rosenberger et al. [10] developed a relationship between the AS viscosity in the TSS concentration function based on AS from nine different MBR plants. The relationship works for concentrations ranging from 2.7 to 46 g/L at 21 °C.

Hasar et al. [11] indicated that the power-law model was the most suitable model to measure AS flow properties in MBRs. They found that shear stress decreased with increasing temperature. The apparent viscosity decreased logarithmically with an increase in the rotary velocity, and increased exponentially when increasing the TSS of the AS. Their results indicated that the temperature and sludge solids concentration were the most critical parameters influencing the flow behavior. In addition, they found the energy demand for sludge transport in the MBR to be elevated due to the high viscosity of conventional activated sludge (CAS) systems.

Garakani et al. [12] compared different viscosity models (i.e., the power-law, Bingham plastic, Herschel–Bulkley, Casson, Sisko, Carreau, and Cross models) to evaluate their performance. They found that the Carreau model provided the best prediction of viscosity over the entire TSS concentration range, while the power-law and Casson models were suitable although less accurate. The power-law model was able to cover all possible shear rates when assuming that the flow is homogeneous, which implies that stress is comparable.

Nevertheless, at low shear rates the shear becomes localized in a small region with a high local shear rate, while the remaining part of the fluid behaves as a solid [13]. The authors of this study proposed a correlation to relate the apparent viscosity to the TSS concentration, shear rate, and temperature. The AS viscosity was found to increase exponentially with the TSS concentration and decrease exponentially when increasing the temperature and shear rate.

Laera et al. [14] and Pollice et al. [15,16] measured the rheological properties of the AS produced from a bench-scale membrane bioreactor at different sludge retention times, corresponding to an increase in TSS. Average rheological properties in steady-state conditions were correlated to the equilibrium biomass concentration by comparing different models. It was found that the Bingham model provided slightly better results than the power-law model in terms of the mean root square error. The two models’ parameters were expressed as functions of the AS TSS concentration, and two expressions for the viscosity were proposed.

Seyssiecq et al. [17] and Mori et al. [18] compared two measuring rheometers, namely, the concentric cylinder (CC) and Double Gap CC (DG-CC) geometries. They found that the CC system is suitable for the characterization of AS, whereas the dimensions of the gaps in the DG-CC geometry are too small, leading to blockage of AS flow. In addition, they investigated the effect of TSS content, finding that an increase in TSS content raises the yield stress value and consistency index while reducing the flow behavior index.

Liu et al. [19] and Radaei et al. [20] developed a CFD model, incorporating an empirically determined rheology model and a porous media model to simulate bubble-induced surface shear in a hollow-fiber MBR. The rheology model was developed for TSS concentrations varying from 3 to 16 g/L. The CFD model was validated using Particle Image Velocimetry (PIV), with the average difference between simulated liquid velocities and PIV measured speeds found to be 5.5%.

Duran et al. [21] analyzed the relationship among AS properties, rheological behavior, and oxygen transfer in a bubble column. They measured the gas hold-up and volumetric oxygen mass transfer and found that the mass transfer coefficient is reduced when the TSS concentration increases.

**Figure 2:**
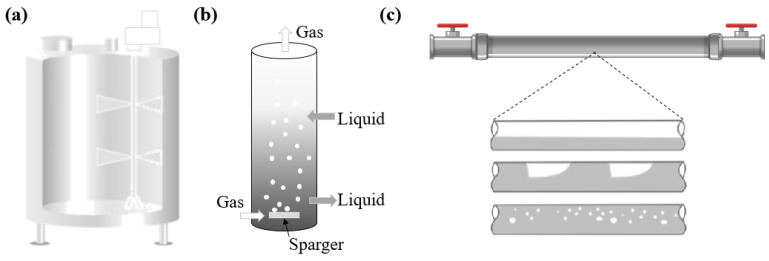
Schemes of (**a**) mixing reactor, (**b**) bubble column reactor, and (**c**) two-phase flow pipe. Adapted from [24,25,26].

Activated sludge comprises aerobic and anaerobic microorganisms, lipids, proteins, colloids, solids, and other compounds is a composition that varies from one wastewater treatment plant to the next. It is an intransparent liquid with a viscosity that is a function of its composition. Based on that, the studies of Moreau et al. [22] and López et al. [23] found that the TSS has more impact on the rheologic behavior than lipids, proteins, or microorganisms, although their review focused only on the suspended solids. The idea behind selecting CMC and XG as surrogates for AS in this study is that when the polymers are mixed with water, the solution is transparent; in this case, knowing the flow behavior and flow consistency indexes allows studies to be performed on the hydrodynamic behavior of MBRs. In the following sections, several applications of CMC and XG based on mixing reactors, bubble columns, and two-phase flow pipelines are discussed; these configurations are illustrated in Figure 2.

Table 1 presents several uses of CMC and XG in mixing reactors along with a variety of subjects related to hydrodynamic behaviors, flow patterns, and non- newtonean fluidized beds. These works contemplate several different models to support their main findngs. Miura et al. [27] developed a correlation for the bed voidage for Newtonian and non-Newtonian liquid–solid two-phase systems, including the drag coefficient correlation for single- and multi-particle systems. Iliuta et al. [28] reached a good agreement between correlations obtained for gas–Newtonian liquid systems and measured the values of the two-phase pressure drop and the total and dynamic liquid hold-up for gas/non-Newtonian liquid systems. Shimizu et al. [29] proposed a model for drop breakage in a stirred tank, including the non-Newtonian effect. Garcia-Ochoa and Gomez [30] developed dimensional correlations expressing the Sherwood number as a function of the Reynolds, aeration, and Weber numbers. Burru and Briens [31] developed a correlation to predict particle–liquid mass transfer coefficients on fluidized beds using an appropriate apparent viscosity to account for the non-Newtonian effect of the liquid.

There are other valuable works comparing the behavior of sludge and CMC/XG. Eshtiaghi et al. [40] identified a transparent liquid mimicking the behavior of thickened digested sludge. Comparing three different liquids revealed that these liquids could function as surrogates for sludge. The sludge’s rheological behavior was modeled using CMC, Carbopol gel, and Laponite clay for high shear rates, short-time flow processes, and where time dependence is dominant, respectively. Bhattacharjee et al. [41], Kennedy et al. [42], Sajjadi et al. [43], Low et al. [44,45], and Xu et al. [46] have all studied the flow regimes that develop when using XG to simulate AS in a mixing vessel operating with a recirculating jet. They observed that the regimes depend on the rheological properties and operating conditions (i.e., the injection velocity). In addition, Markis et al. [47] studied how the rheology of sludge changes over time in order to predict the performance of pumping and mixing systems.

For the case of two-phase flow in pipes, Table 2 presents several works that applied CMC or XG. This table mainly covers velocity profiles or pressure drop for upward and downward vertical, inclined, and horizontal pipes. As in the case of reactors, these works developed different correlations, as in the research of Biswas and Das [48]. Based on its operating and geometric parameters, they correlated the experimental data on the two-phase frictional pressure drop using a friction factor method in dimensionless groups. This was developed as an empirical correlation to calculate the two-phase friction factor. Dziubinski and co-workers identified that correlations for void fraction and frictional pressure drop for two-phase flow cannot be directly used for non-Newtonian fluid, as corrections must be applied in order to account for non-Newtonian behaviors.

Majumder and co workers have developed different empirical correlations related to this subject. Majumder et al. [65] proposed a correlation for void fraction in vertical two-phase slug flow considering the liquid’s non-Newtonian behavior as a function of the modified Reynolds number based on apparent viscosity and the liquid property group. Majumder et al. [66,67] developed a correlation for predicting pressure drop in a downflow bubble column operating with a non-Newtonian liquid. The correlation considers bubble formation, drag force, wettability, and interfacial shear stress.

Finally, regarding bubble columns, Table 3 shows several applications associated with CMC or XG, including gas hold-up and bubble behavior based on experimental analyses. As in the previous cases, these works developed useful correlations. Haque et al. [68] obtained a correlation between the void fraction in the function of the viscosity and the bubble column’s diameter. Dapelo et al. [69] developed a novel Euler–Lagrangian model in CFD for gas mixing in anaerobic digestion. The model was validated with lab-scale data with PIV. CMC was used as the non-Newtonian fluid to validate the model in CFD. They found good agreement between the CFD results and experimental measurements.

## 3. Comparison of Rheological Parameters

### 3.1. Proposed Assessment: Seeking for Insights and Trends

**Figure 3:**
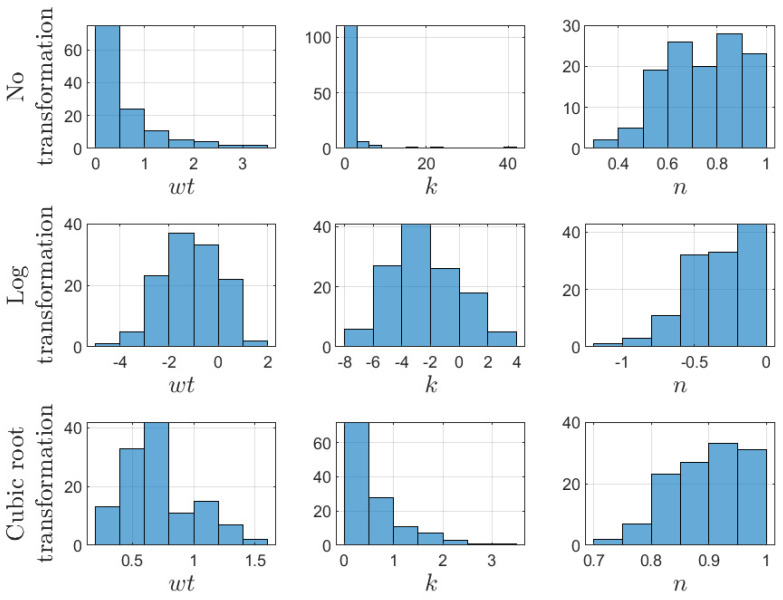
Histograms of wt, *k*, and *n* for the CMC records, with logarithm and cubic root transformations.

The rheological parameters of the CMC, XG, and AS compounds were analyzed based on their reported flow consistency (*k*) and flow behavior (*n*) indexes. For this purpose, linear and non-linear regressions were contemplated, considering as main predictors the concentration wt (%) and the temperature in °C. Logarithmic and cubic root transformations were used, as certain features such as *k* and *T* had more comprehensive ranges (i.e., greater than 10:1); see Figure 3. These types of transformations have been successfully applied in previous works [82,83] to help stabilize the variance of the data [84].

Let *Y* denote the response and $X_{i}$ stand for the variables evaluated. The logarithmic regression then has the general form shown in Equation (6) for the linear case, which is equivalent to a general power relation. Here, $\beta_{0}$ and $\beta_{i}$ are the intercept and regression coefficients obtained from a least-squares approach, while ϵ is the associated error.

(6) $$\log Y=\beta_{0}+\sum_{i}\beta_{i}\log X_{i}+\epsilon$$

A similar expression can be obtained for the cubic root transformation for both the response and the variables evaluated, as follows:

(7) $$Y_{13}:=\sqrt[{3}]{Y}=\beta_{0}+\sum_{i}\beta_{i}{X_{13}}_{i}+\epsilon$$

The model fit was evaluated based on two well-known numerical measures: the Residual Standard Error (RSE) and the adjusted $R^{2}$ statistic. The former estimates the standard deviation of the error using the Residual Sum of Squares (RSS), while the latter is a measure of the linear relationship between the variables and the response. Non-linear regressions were considered and included as well; $R_{adj}^{2}$ was estimated in these cases using the predicted and the actual response observations.

In addition, the Akaike and Bayesian Information Criteria (AIC and BIC) were implemented; while these two well-known selection criteria may seem meaningless in this context, they are commonly used to compare competing models or regressions [85]. AIC ranks the information loss and the unaccountable information. In contrast, BIC is based on a log-likelihood function incorporating the deviance of the model fit and penalizing for additional parameters [86]. Both criteria prefer those models with the lowest results regardless of the sign of the result. Additional information about these selection criteria can be found elsewhere; see for instance [87].

In this work, the modified versions of the AIC and BIC were implemented because they have been recommended for small samples, i.e., $n_{obs}/k^{*}<40$, where $k^{*}=p+1$, *p* denotes the number of fitted parameters, and $n_{obs}$ is the number of observations for the regression [88]. These modified versions are shown below:

(8) $$\begin{array}{c} AIC_{c}=\left\{n_{obs}\left[\log\left(\frac{RSS}{n_{obs}}\right)\right]+2k^{*}\right\}+n_{obs}+n_{obs}\log\left(2\pi \right)+\frac{2k^{*}\left(k^{*}+1\right)}{n_{obs}-k^{*}-1}\\ BIC_{c}=n_{obs}\left[\log\left(\frac{RSS}{n_{obs}}\right)\right]+k^{*}\log n_{obs}+n_{obs}+n_{obs}\log\left(2\pi \right) \end{array}$$

In the following sections, the results from the CMC, XG, and AS datasets are described in greater detail.

### 3.2. CMC Regression Results

After a thorough review, it was possible to extract the rheological parameters for CMC records associated with weight concentration, flow indexes (i.e., *k*, *n*), shear rate, temperature, geometry, and the implemented rheometer from 37 different authors; Table 4 summarizes this information. This table depicts varied ranges of applications for each feature, highlighting where certain authors did not report the experimental temperature. Because the intention was to build a regression model using the weight concentration and the temperature as variables, a total of 129 records were used from 31 different authors for this analysis.

#### 3.2.1. Prediction of *k* as a Function of wt and *T*

The first assessment focused on the regression between wt and *k*, bearing in mind that they have the highest linear correlation. Table 5 displays the obtained results using the original (dSet), logarithmic (log(dSet)), and cubic root ($dSet^{1/3}$) transformed datasets for different linear, power-law, and exponential regressions. These non-linear expressions were implemented following the types of relations mentioned in Equations (4) and (5).

Recall that the best regression is the one with the highest $R_{adj}^{2}$ and lowest RSE, AICc, BICc, and *p*-Value. Table 5 indicates that applying the logarithmic or cubic root transformation helps to describe the variance and main changes of *k* in terms of wt, with the cubic root approach being the one that results in a lower RSE, $AIC_{c}$, and $BIC_{c}$ in every case. Note that the *p*-values were statistically significant and that all these regressions used two predictors in order to avoid biased penalization results for the information criteria indicators. It can additionally be noted that slightly better results were produced when not including an intercept in the regression for the linear case; however, the transformed datasets triggered the worst $R_{adj}^{2}$ results. For the case of non-linear regressions, the exponential regressions exposed improved results compared to the power-law expressions, although it should be noted that it is not feasible to fit a power function to data where the independent variable (i.e., wt) has non-positive values because of the logarithmic transformation.

**Figure 4:**
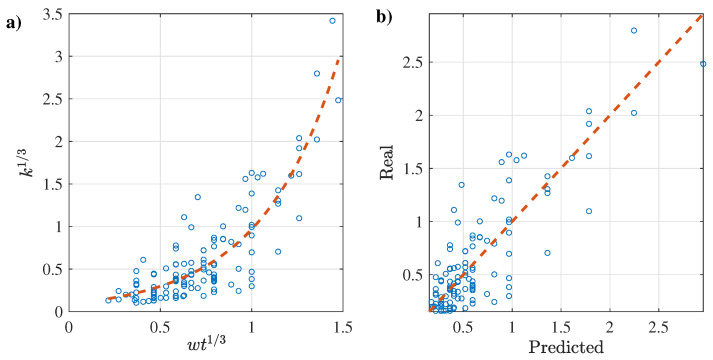
(**a**) $k^{1/3}$ vs. $wt^{1/3}$ best fitting results and (**b**) predicted vs. actual $k^{1/3}$ results.

Based aforementioned results, the best regression implemented the exponential regression with the cubic root transformation, as follows:

(9) $$k_{13}=0.09163\exp\left(2.35722\cdot wt_{13}\right)$$

where the 95% coefficient intervals of these coefficients are (0.06783–0.11544) and (2.13453–2.57992) for the first and second coefficient, respectively. The fitting results are depicted in Figure 4, where it can be noted that there is a good agreement between the predicted and actual reported data.

**Figure 5:**
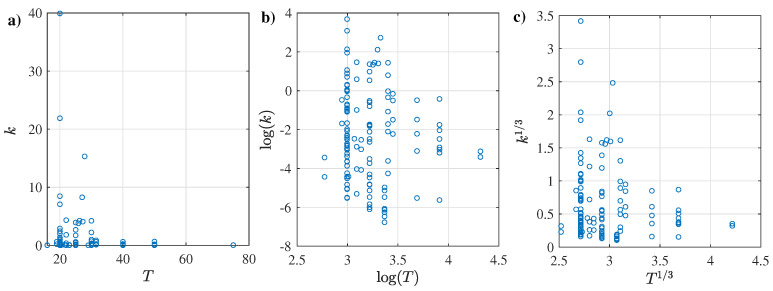
Scatter plot of (**a**) *k* vs *T*, (**b**) log(k) vs. log(T), and (**c**) $k^{1/3}$ vs. $wt^{1/3}$.

Following a similar procedure as described above, the regressions between the flow consistency index *k* based on the temperature *T* were evaluated, obtaining the results shown in Table 6. These results suggest that based solely on the $R_{adj}^{2}$ results, none of the regressions reach a good fitting between *k* and *T* except for linear regression using the cubic root transformation without an intercept. Considering the other regression descriptors, an exponential regression with the same transformation could be recommended, although it has poor prediction behavior. This behavior can be explained by the fact that the reported temperature follows a scattered or dispersed pattern regarding the flow consistency index, as depicted in Figure 5 for the original dataset and both transformations.

**Figure 6:**
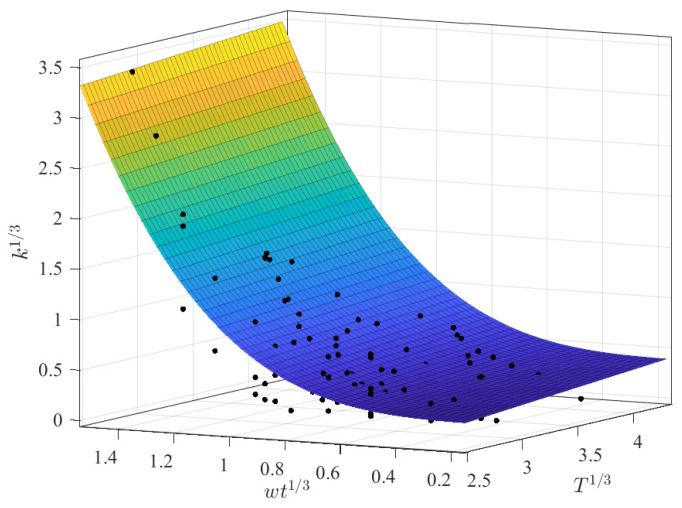
Best fitting results for the CMC dataset with $k^{1/3}$ as a function of $wt^{1/3}$ and $T^{1/3}$.

A multivariate regression approach was implemented using polynomial and different power and exponential expressions based on the previous results from the regressions between *k* vs. wt and *k* vs. *T*. The best regression results are summarized in Table 7, with the result using a power-law expression with wt and a linear relation with *T* highlighted (Figure 6). It can be noted that the prediction capabilities slightly decreased from the approach using only wt, which was expected due to the dispersed pattern with temperature. For this case, all parameters were reported as extremely significant (*p*-Value < 2 × 10^−5^) with the following coefficients:

(10) $$k_{13}=0.74957\cdot {\left(wt_{13}\right)}^{3.283}+0.07617\cdot T_{13}$$

Although other polynomial alternatives were contemplated as well, including additional second or superior order coefficients did not improve the prediction capabilities.

#### 3.2.2. Prediction of *n* as a Function of wt and *T*

The subsequent analysis implemented *n* as the response and wt and *T* as predictor variables. Different linear, power-law, and exponential expressions were tested to describe *n* in terms of wt, obtaining the results summarized in Table 8. This table shows worse results regarding $R_{adj}^{2}$ than in the case with *k*, although with lower residual errors because of the range of the flow behavior index. Concerning the regression results, the best regression results were obtained using the exponential behavior with the cubic root transformation thanks to its lower $AIC_{c}$, $BIC_{c}$, RSE, and an $R_{adj}^{2}$ close to the highest determined:

(11) $$n_{13}=0.9983\exp\left(-0.1472\cdot wt_{13}\right)$$

**Figure 7:**
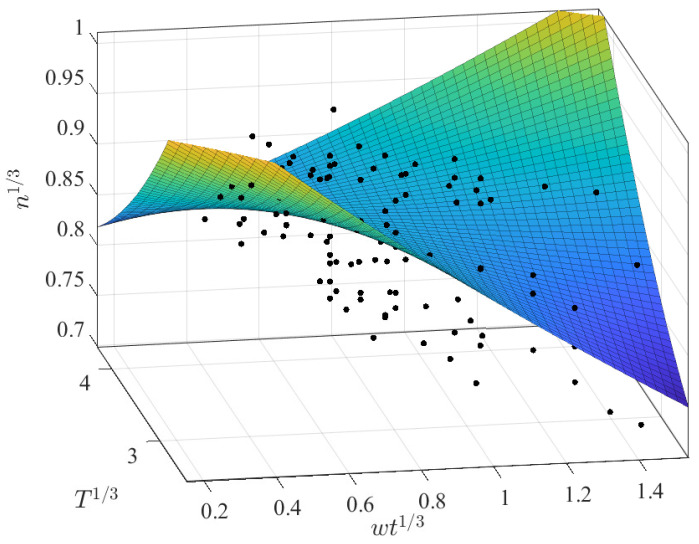
Best fitting results for the CMC dataset with $n^{1/3}$ as a function of $wt^{1/3}$ and $T^{1/3}$.

Different regressions were assessed for *n* vs. *T*; however, as before, none of the regressions obtained good prediction results regarding the temperature. We were only able to determine that implementing an intercept in the linear regressions increases prediction capability, and that the “best” regression result occured when applying the cubic root transformation. A multivariate approach was thus implemented using this transformation. In this regard, better results were reached with a second-order polynomial, although these were still not as good as those reported previously for the flow consistency index (RSE 0.0313, $R_{adj}^{2}$ 0.3683). The final fit is shown below and displayed in Figure 7. In this case, note that the prediction capabilities are again very similar to those using only wt as the predictor.

(12) $$n_{13}=2.11644-1.78834\cdot wt_{13}-1.54229\cdot T_{13}+{\left(wt_{13}\right)}^{1.04721}+{\left(T_{13}\right)}^{1.14690}+0.21176\cdot wt\;T$$

#### 3.2.3. Prediction of *k* as a Function of *n*

**Figure 8:**
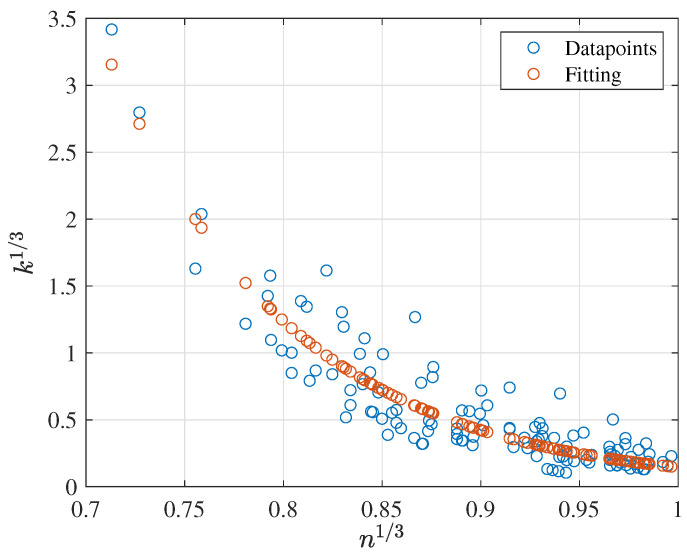
Best fitting results for the CMC dataset: $k^{1/3}$ vs. $n^{1/3}$.

As a final result for the CMC dataset, different regressions between *k* as a function of *n* were assessed. In this regard, it was found that the results were better for the cubic root transformation with an exponential regression again. For this purpose, an RSE of 0.0805 and an equivalent 0.8045 $R_{adj}^{2}$ highlight it as a good fitting, as can be confirmed from Figure 8. The final coefficients are displayed below:

(13) $$k_{13}=6681.2041\exp\left(-10.7412\cdot n_{13}\right)$$

### 3.3. XG Regression Results

It was possible to extract the rheological parameters for XG records associated with weight concentration, flow indexes (i.e., *k* and *n*), shear rate, temperature, geometry, and the implemented rheometer from 13 different authors; Table 9 summarizes this information. This table indicates that several authors did not report the flow consistency index or temperature; for this analysis, 43 records were available from nine authors.

#### 3.3.1. Prediction of *k* as a Function of wt and *T*

Following a similar procedure as the one implemented for the CMC dataset, we determined a set of different regressions for *k* based on wt and *T*. For this purpose, the results for *k* vs. wt are shown in Table 10, whereas no adequate regression could be determined using only temperature as the predictor.

**Figure 9:**
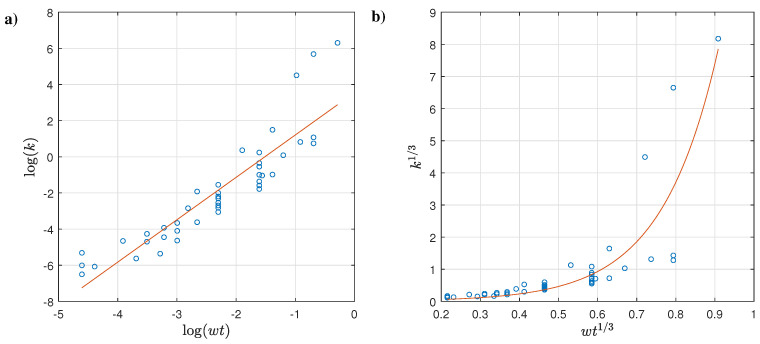
Best (**a**) linear and (**b**) non-linear regressions for the XG dataset.

Table 10 indicates that the best regression results correspond with linear regression with a logarithmic transformation and an exponential expression with a cubic root transformation. Figure 9 displays the observations and fittings in both cases in order to indicate how different these predictions are. Based on this figure, it is clear that the three highest records of the flow consistency index *k* play a significant role in both regressions. These observations were reported by Japper-Jaafar et al. [98], with *k* values of 90.6969, 294.0987, and 546.7029. This figure indicates that these “outliers” may affect the exponential regression more significantly than the linear regression with the logarithmic transformation.

**Figure 10:**
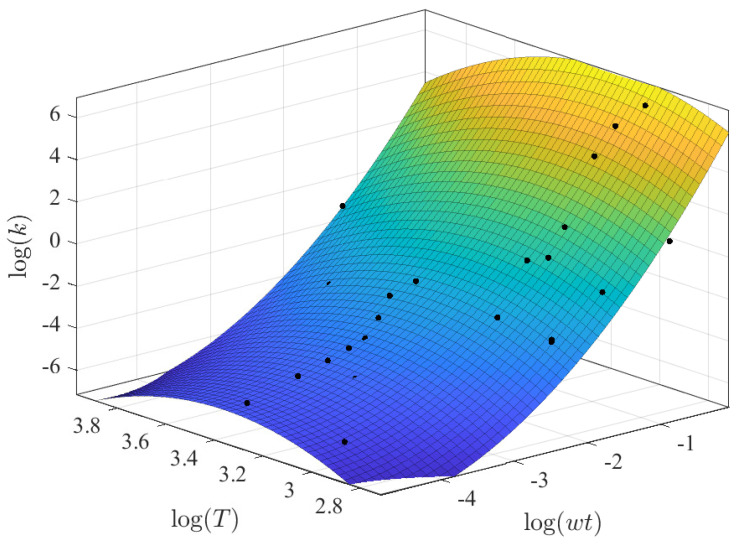
Best fitting results for the XG dataset with log(k) as a function of log(wt) and log(T).

In addition to the results mentioned above, different multivariate regressions were evaluated, considering only the cases with the logarithmic and cubic root transformations for the *k* vs. wt results; Table 11 presents the main results. This table shows general predictions of the flow consistency, especially for the combined power-law approach using $dSet^{1/3}$ and the second-order polynomial with log(dSet). Among them, the last regression is highlighted due to its high $R_{adj}^{2}$, which was not surprised considering the results from the univariate regressions with wt and *T* showing a good agreement in both predictors, as displayed in Figure 10.

The results for this polynomial approach are as follows:

(14) $$\begin{array}{cc} \log(k)= & -50.5387+6.8966\cdot \log\left(wt\right)+36.5891\cdot \log\left(T\right)+0.5506\cdot \log{\left(wt\right)}^{2}\\ \; & -5.7660\cdot \log{\left(T\right)}^{2}-0.4682\cdot \log\left(wt\right)\;\log\left(T\right) \end{array}$$

**Figure 11:**
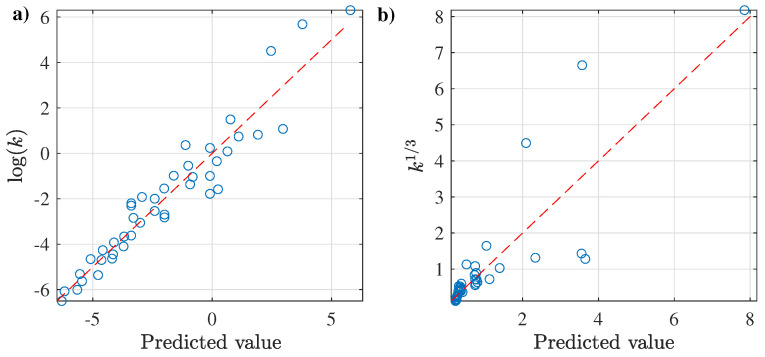
Prediction results for the XG dataset of (**a**) polynomial regression in Figure 10 and (**b**) the best power combined approach.

In order to compare the prediction capabilities from both approaches in more detail, the prediction results were determined, and are depicted in Figure 11, where it can be clearly seen that the polynomial approach outperforms the one using the combined power regression. The latter approach has poor prediction for $k^{1/3}$ greater than 1.5. At the same time, the former regression describes even the data from the Japper-Jaafar et al. [98] outliers in a more acceptable way.

#### 3.3.2. Prediction of *n* as a Function of wt and *T*

Regarding the regression for *n* in terms of wt and *T*, we obtained the results shown in Table 12, whereas no adequate regression could be determined using only temperature as the predictor. As in the CMC dataset, the results in Table 12 indicate that wt and *n* are less correlated than in the case of *k*, reaching more dispersed predictions and having lower prediction capabilities.

The regression results show that the best predictions are obtained using the cubic root transformation with the power and exponential expressions, with $R_{adj}^{2}$ lower or equal to 0.40 in each case. After implementing multivariate regression with both wt and *T* predictors, the results rise until 0.56 for a second-order polynomial, as depicted in Table 13. The obtained coefficients from this fit are presented in the following expression:

(15) $$n_{13}=-2.175-3.0871\cdot wt_{13}+2.6136\cdot T_{13}+1.0075\cdot {\left(wt_{13}\right)}^{2}-0.4994\cdot {\left(T_{13}\right)}^{2}+0.6464\cdot wt_{13}\;T_{13}$$

**Figure 12:**
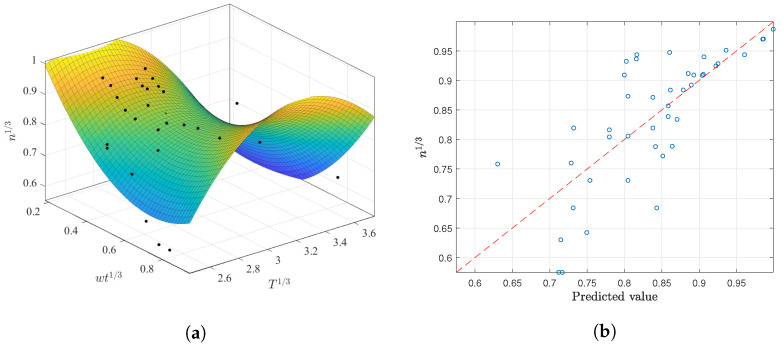
Best polynomial regression of $n_{13}$ in terms of $wt_{13}$ and $T_{13}$ for the XG dataset: (**a**) fitting results and (**b**) prediction capabilities.

Figure 12 shows the obtained fitting and prediction capabilities with the XG records. This figure suggests that the obtained fit has better results for values greater than $n_{13}=0.75$ and is more accurate for $n_{13}>0.85$. Unfortunately, the number of recollected observations is not as large as in the datasets for CMC and AS, which could affect the final descriptors and principal trends with the flow behavior.

#### 3.3.3. Prediction of *k* as a Function of *n*

**Figure 13:**
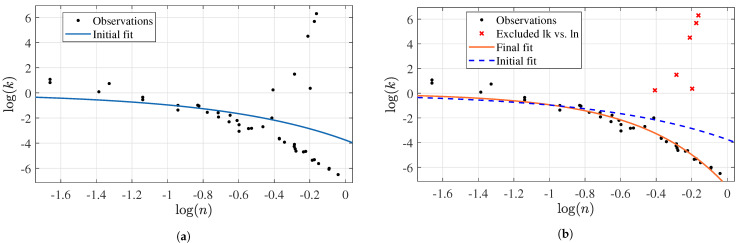
Exponential regression results of log(k) and log(n) for the XG dataset: (**a**) initial fitting and (**b**) fitting after filtering.

Our final analysis using the XG dataset contemplated the relationship between the flow consistency and behavior indexes. For this purpose, linear and non-linear approaches were evaluated, reaching unsatisfactory results with most regressions because of tentative “outlier behavior” in a few records reported by Japper-Jaafar et al. [98]. These records have the highest flow consistency index at 1.4391, 1.2685, 4.4474, 90.6969, 294.0987, and 546.7029. Figure 13a shows the obtained fit, which is clearly biased by the abovementioned records. In this case, the reported RSE was 2.8336 and the $R_{adj}^{2}$ was 0.0853. By excluding these six records from the analysis, the prediction capabilities would be significantly improved (RSE 0.4966 and $R_{adj}^{2}$ of 0.9421), as illustrated in Figure 13b, where the orange line matches this observation quite precisely. The best performance results follow an exponential behavior. This pattern is similar to the case of the CMC dataset, except for the concavity, with the CMC case decreasing with positive concavity and the XG with negative concavity:

(16) log(k)=−7.565exp(2.09·log(n))

### 3.4. AS Regression Results

Finally, the rheological parameters for AS records associated with weight concentration, flow indexes (i.e., *k* and *n*), shear rate, and temperature were extracted from 17 different authors, of which 11 authors reported all the information in 148 records; Table 14 summarizes this information. In this case, three records reported by Khalili et al. (2011) were not included, as they different from the rest of the collected data by more than an order of magnitude.

#### 3.4.1. Prediction of *k* as a Function of wt and *T*

As reported for the CMC and XG datasets, different regression alternatives were found for wt and no satisfactory results were found using the temperature. From Table 15, the power and exponential expressions using the cubic root transformation are again highlighted due to their lower RSE and higher $AIC_{c}$ and $BIC_{c}$ results. In this case, the exponential approach was selected, obtaining the following coefficients:

(17) $$k_{13}=0.09477\exp\left(1.79963\cdot wt_{13}\right)$$

Regarding the combined multivariate expression, the best results were obtained from a second-order polynomial as compared to the combined power and first-order approaches (Table 16). The coefficients for this fit are shown below. The more significant coefficients were the intercept, second-order coefficients, and coefficient of the temperature:

(18) $$k_{13}=45.1543-8.9259\cdot wt_{13}-29.2344\cdot T_{13}+2.5978\cdot {\left(wt_{13}\right)}^{2}+4.9828\cdot {\left(T_{13}\right)}^{2}+1.5731\cdot wt_{13}\;T_{13}$$

**Figure 14:**
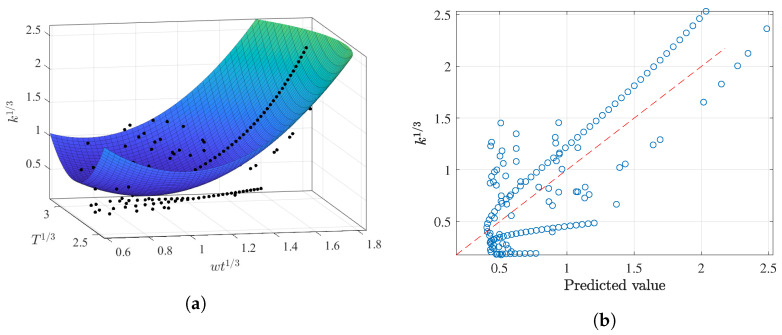
Best polynomial regression result of *k* in terms of wt and *T* for the AS dataset: (**a**) fitting results and (**b**) prediction capabilities.

Figure 14 depicts the obtained results from this multivariate approach. Figure 14a shows that certain observations around $wt_{13}$ of 0.8 could not be accurately predicted, reporting low *k* values. Furthermore, it should be noted that the predicted surface lies between two sets of parallel observations, as illustrated in Figure 14b. These dispersed observations complicate the final predictions and indicate just how complicated it is to precisely predict these parameters.

#### 3.4.2. Prediction of *n* as a Function of wt and *T*

The results for both wt and *T* were not completely satisfactory. The best regression of *n* vs. wt contemplates an exponential form without any transformation, with an $R_{adj}^{2}$ of 0.23 and RSE of 0.09. For the case of temperature, the results are “better” when using an intercept and a cubic root transformation.

Regardless of the bad results from the univariate cases, a multivariate approach was determined for different expressions, as shown in Table 17. In this case, the best results are associated with a second-order polynomial using the cubic root transformation with the following coefficients:

(19) $$n_{13}=-2.9993-2.7984\cdot wt_{13}+1.8567\cdot T_{13}-0.5381\cdot {\left(wt_{13}\right)}^{2}-0.2338\cdot {\left(T_{13}\right)}^{2}-0.6494\cdot wt_{13}\;T_{13}$$

**Figure 15:**
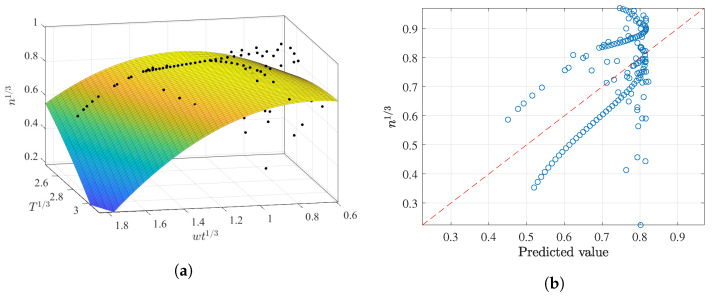
Best polynomial regression result of *n* in terms of wt and *T* for the AS dataset: (**a**) fitting results; (**b**) prediction capabilities.

The obtained surface is depicted in Figure 15a, while the predicted values are compared with the reported $n_{13}$ in Figure 15b. It is not surprising that the surface would lie between the two sets of parallel observations, as occurred in the *k* multivariate results. This multivariate regression may not be recommended for $n_{13}>0.8$ in light of their greater prediction residuals.

#### 3.4.3. Prediction of *k* as a Function of *n*

**Figure 16:**
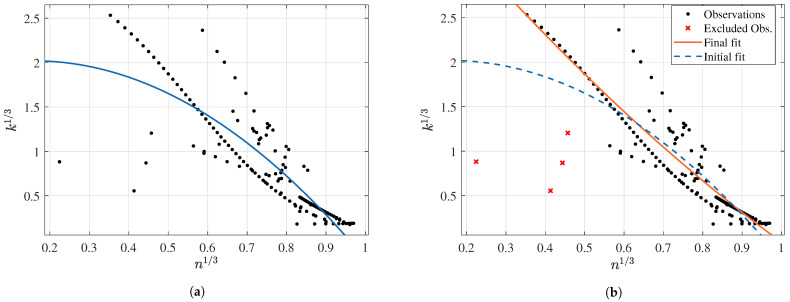
Exponential regression results of $k_{13}$ and $n_{13}$ for the AS dataset: (**a**) initial fitting and (**b**) fitting after filtering.

Finally, the behavior between *k* and *n* was evaluated for the three datasets, obtaining better results with the cubic root transformation. As occurred with the XG dataset, the observations have tentative outliers that drastically affect any regression between these two features. Figure 16 illustrates this behavior before and after excluding the four outlier observations. In the first case, using an exponential regression with two components reached a fitting with $R_{adj}^{2}$ 0.7277 and RSE 0.3161. When removing the four outliers, the prediction capabilities dramatically improved to $R_{adj}^{2}$ 0.8513 and RSE 0.2365. The final expression is then as follows:

(20) $$k_{13}=-0.5626\exp\left(1.019\cdot n_{13}\right)+5.144\exp\left(-1.217\cdot n_{13}\right)$$

## 4. Conclusions

Viscosity plays a significant role in hydrodynamic regime, oxygen transfer, and mass transport in different systems, MBRs among them, consequently influencing system performance. Therefore, an accurate viscosity relationship as a function of concentration is required. In this regard, Activated Sludge (AS) is vital in describing the behavior of MBR. Its aerobic and anaerobic microorganisms, lipids, proteins, colloids, solids, and other compositions vary from one wastewater treatment plant to the next. AS is an intransparent liquid. This work reviews the possibility of using two non-Newtonian polymeric liquids, i.e., carboxymethyl cellulose (CMC) and xanthan gum (XG), to mimic the behavior of AS inside an MBR. Therefore, rheological parameters directly related to the fluid viscosity were studied. After a thorough survey of the literature, different rheological parameters, principally the flow consistency (*k*) and behavior (*n*) indexes, were found for CMC, XG, and AS based on the concentration and temperature, in turn allowing the fluid viscosity in the MBR to be described.

The differences found in the data for the flow behavior and flow consistency index can be attributed to the following:

1. Research using these different compounds does not focus on the determination of rheological properties, except for the work of Benchabane and Bekkour [90] and that of Ghannam and Esmail [94]. All other studies used the non-Newtonian compounds to determine the behaviors of specific systems. Therefore, no attention was paid to the procedure by which the viscosity measurements were obtained.
2. Different rheometers/geometries were used for the viscosity measurements (i.e., concentric cylinders, cone-and-plates, double gaps, and tubular), which produced different results.
3. Various experimental protocols were implemented.
4. The chemicals used were from different chemical manufacturers.

Different curves (shear rate vs. viscosity/shear stress) are obtained because each device and procedure seems to modify the polymer structure. It is challenging to characterize the viscosity of non-Newtonian liquids regarding concentrations, and no single model correctly predicts the viscosity. Therefore, it is recommended that the solutions be characterized based on their rheological properties (flow behavior and flow consistency index), not their concentrations, as it is impossible to compare CMC, XG, and AS solutions based on the values of their concentrations.

This work has presented several insights about both indexes based on different linear and non-linear regressions when using the concentration and temperature as variables. Good predictions could be obtained, especially for the CMC recollected data, although the data sample was small. The results obtained here aim to support decision-making processes related to the design and operation of MBRs while considering the principal role of viscosity.

## Figures and Tables

**Table 1:** Applications of CMC and XG in mixing reactors.

Author	Main Subject	Main Contribution
Abukhalifeh et al. [32]	Effect of non-Newtonian flow behavior on the turbulent bed contractor’s hydrodynamic characteristics on a fluidized bed.	High apparent viscosity solutions result in a more densely fluidized bed; the liquid hold-up increases with the high viscosity for different liquid flow rates.
Lali et al. [33]	Experimental particle velocities (glass and stainless steel) in viscous non-Newtonian liquids on fluidized beds.	Modified Reynolds number (apparent viscosity) is sufficient to account for increased shear at high Reynolds numbers.
Miura and Kawase [34,35]; Miura et al. [27]	Non-Newtonian liquid–solid two-phase in fluidized beds.	Bed voidage augmented with an increase in the liquid velocity. At a given liquid velocity, the magnitude decreases as particle size grows and is more significant as viscosity or viscous non-Newtonian flow behaviors increased.
Aydin and Larachi [36]	Effects of temperature and pressure on the hydrodynamics of trickle-bed reactors with Newtonian and non-Newtonian liquids (flow regime transition, pulse velocity, two-phase pressure drop, liquid hold-up, and liquid axial dispersion coefficient).	The trickle-to-pulse flow regime transition boundary shifts to higher fluid velocities with increased temperatures, and pulse velocity increases with temperature.
Iliuta et al. [37]; Iliuta and Thyrion [38]; Aydin and Larachi [36]; Iliuta et al. [28]	Hydrodynamics of two-phase down- and up-flow through fixed beds with porous particles (flow regimes, two-phase pressure drops, and liquid hold-ups).	The presence of non-Newtonian liquids strongly influences them. With highly viscous non-Newtonian liquids, the two-phase down-flow hydrodynamic characteristics are close to those of two-phase up-flow. Axial dispersion is an actual cause of possible hydrodynamic effects that can impact reactor performance.
Shimizu et al. [29]	Drop size distributions in a stirred tank with a non-Newtonian liquid (CMC and XG) with dispersion process without coalescence.	The non-Newtonian characteristics of the continuous phase caused an increase in the drop size at low impeller speeds.
Garcia-Ochoa and Gomez [30]	Oxygen transfer rate in Newtonian and non-Newtonian fluids in a stirred tank.	The volumetric mass transfer coefficient was determined to change many variables (i.e., superficial gas velocity, stirrer speed, and the liquid’s rheologic properties).
Sindall et al. [39]	Positron emission particle tracking (PEPT) to visualize the flow patterns on an anaerobic digester fitted with mechanical or gas mixing systems.	Sewage sludge and a transparent synthetic sludge (CMC) calculated the digester’s velocity profiles. PEPT was used to accurately validate CFD models, which could be used to optimize flow patterns for biogas production.

**Table 2:** Applications of CMC and XG in two-phase flow in pipes.

Author	Main Subject	Main Contribution
Das and Biswas [49]; Biswas and Das [48]	Two-phase pressure drop in vertical tubes and helical coils using gas–non-Newtonian liquid.	Analysis of the effects of the gas flow rate, liquid flow rate, concentrations of CMC, and coil diameter on the pressure drop.
Dziubinski [50,51]; Dziubinski et al. [52]; Dziubinski and Marcinkowski [53]; Fidos et al. [54]	Pressure drop and void fraction for two-phase flow in vertical pipes using non-Newtonian liquids.	The generalized Reynolds number resulting from the power-law (non-Newtonian) liquid model cannot be used to determine the Taylor bubble rising velocity.
Jinming and Jingxuan [55]; Xu et al. [56]; Xu and Wu [57]; Xu et al. [58]	Upward and downward two-phase flow in horizontal, inclined, and vertical pipes using non-Newtonian liquids.	Measurement of void fraction and pressure drop to develop a void fraction and pressure gradient model validated against a large set of available experimental data over a wide range of operating conditions, fluid characteristics, and pipe diameters.
Yang et al., [59] and Zhang et al. [60]	Two-phase flow of nitrogen/non-Newtonian fluid (CMC and XG) in non-circular and circular microchannels, respectively.	Flow pattern maps based on observation.
Fan et al. [61]	Flow field around two parallel bubbles rising from two uniform orifices submerged in non-Newtonian liquid using Particle Imaging Velocimetry (PIV).	The fluid velocity in front of and behind two bubbles increases with gas flow rate due to the CMC solution’s shear-thinning effect. However, the speed decreases with increasing solution concentration due to the rising drag force acting on the bubbles.
Sousa et al. [62,63]	Flow around a single Taylor bubble rising in non-Newtonian solutions using PIV and shadowgraphy (the bubble interface’s correct position and different shapes of bubbles)	Flow around the nose of the bubbles was similar to that around all the bubbles. Velocity profiles in the liquid film around the bubble and the tail (wake region) formed different structures. This result was attributed to the increase in viscosity, and the wake flow pattern varied from turbulent to laminar. In addition, a negative wake was observed for solutions with higher polymer concentrations.
Araujo et al. [64]	A CFD study about the rise of individual Taylor bubbles through CMC.	Detailed predictions of viscosity and shear rate distributions that are unattainable with experimental techniques. Application of CFD tools to describe this system was successfully validated against experimental data obtained from photography and PIV/PST measurements of individual Taylor bubbles.

**Table 3:** Applications of CMC and XG in bubble columns.

Author	Description	
Wachi et al. [70]	Gas hold-up and liquid circulation rates in a bubble column with Newtonian and non-Newtonian liquids.	Fluid properties (i.e., viscosity and surface activity) affected gas hold-up between riser and downcomer and consequent liquid circulation. High viscosity and low surface tension of Newtonian liquids (glycerol and ethanol, respectively) reduced the circulation rate compared to water, whereas non-Newtonian fluids (CMC) increased liquid velocities.
Deng et al. [71]	Gas hold-up, bubble behavior (size and shape), interfacial area, and gas–liquid mass transfer in a bubble column.	Increasing the bubble velocity or CMC concentration led to a broader bubble size distribution. The mass transfer coefficient increased with increasing bubble velocity and decreasing CMC concentration.
Fransolet et al. [72]	Experimental analysis of fluid rheology’s influence on the gas flow pattern in a bubble column reactor. Bubble size distributions were measured using parietal pressure probes; the gas hold-up and air distribution were determined by 2D electrical resistance tomography (ERT) images of the gas distribution within the bubble column.	The gas flow pattern within the column was different for Newtonian and non-Newtonian fluids. Gas hold-up values decreased with increasing liquid viscosity, reaching a plateau. A homogeneous flow regime at low gas velocities tended to disappear when viscosity increased.
Babaei et al. [73]	Electrical resistance tomography (ERT) measurements as a non-intrusive approach to examining the influence of MLSS concentration and aeration intensity on gas hold-up within the bioreactor.	Gas hold-up was a determinant factor regarding the efficiency of AS processes. An increase in MLSS concentration led to an initial increase followed by decreasing variation in overall gas hold-up.
Bajon Fernandez et al. [74]	Gas-to-liquid mass transfer in digested sludge (shear-thinning fluid) and the impact of apparent viscosity on process performance using a pilot-scale absorption bubble column for Newtonian and shear-thinning fluids with different superficial gas velocities.	A reduction of mass transfer efficiency with increasing viscosity was observed. Conditions imitating the rheological variability of anaerobically digested sewage sludge were included with CMC. Rheology should be accounted for in the design of mass transfer systems to avoid reductions in process performance and active volume.
Passos et al. [75]	Observation of organic surface-active agents’ effect in bubble column equipped with a porous sparger containing a non-Newtonian liquid (XG).	The diameter of the bubbles decreased as a function of the gas flow rate.
Cao et al. [76]	Use of XG as surrogate fluid for digested sludge due to its excellent optical clarity and stability.	The rheological behavior of XG was similar to sludge; it followed the same trend in the rheological curves, with similar curvatures.
Bobade et al. [77,78,79]	Gas injection influence on the rheological properties of XG was used to model sludge.	The XG flow curve showed negligible change in apparent viscosity in the flow region as the gas injection flow rate increased. However, a creep test strengthened the molecular structure in the linear viscoelastic region. This means that XG is unsuitable as a model fluid for the sludge under gas injection below the yield stress point.
Dong et al. [80]	Experimental examination of mass transfer performance in CMC.	Liquid phase rheology had a significant effect on gas–liquid mass transfer performance. CMC’s volumetric mass transfer coefficients were much smaller than those of water.
Bohm et al. [81]	Bubble swarm behavior in a flat sheet MBR. The channel depth, superficial liquid and gas velocity parameters, and continuous phase viscosity were varied. Along with water, XG was used as a non-Newtonian liquid to investigate the rheology of actual wastewater sludge.	Significant influence on the rheology was found for both measured properties, indicating that the results gained in water are not transferable to entire systems.

**Table 4:** Different rheological parameters for CMC available in the literature.

Concentration Range (wt.%)	*k* Range (*Pa s^n^*)	*n* Range (–)	Shear Rate Range (s^−1^)	*T* (°C)	Geometry	Rheometer	Ref.
0.20–1.00	0.0041–0.0266	0.90–0.98	10–1200	20	NM	AR 2000-TA Instruments	[32]
0.90–3.20	3.7900–15.3100	0.61–0.79	0.1–200	25–28	CC	Bohlin CVO-100	[89]
0.25	0.0330–0.0720	0.66–0.71	0–1000	25–75	NM	Advanced Rheometric Expansion System (ARES)	[36]
0.20–3.00	0.0584–39.9221	0.36–0.62	0–1000	20	CP	AR 2000-TA Instruments	[90]
0.02–0.08	0.0142–0.7112	0.60–0.90	NM	30	T	pipeline viscometer	[48]
0.05–0.20	0.0040–0.0520	0.72–0.91	NM	NM	NM	NM	[91]
0.50–0.70	0.0119–0.0322	0.92–0.99	0–1000	16	DG	RHEOTEC RC 20	[92]
0.20–0.40	0.0135–0.1211	0.67–0.87	NM	25	NM	Haake Rotovisco	[31]
0.2–0.8	0.054–1.336	0.619–0.805	100–500	35	CC	AR1000	[69]
0.05–0.10	0.1080–0.8530	0.65–0.80	NM	31.5	T	pipeline viscometer	[49]
0.20–0.45	0.0065–0.0973	0.73–0.96	NM	NM	NM	NM	[71]
0.3–0.6	0.043–0.3	0.7–0.84	NM	20	CC	Brookfield R/S	[80]
0.50–1.10	0.1623–3.9265	0.50–0.73	5–1230	25	NM	NM	[93]
1.00	0.3380	0.83	NM	NM	NM	NM	[51]
0.5–1.5	0.024–2.313	0.309–0.633	0.001–1000	25	CC	SR2000	[40]
0.35–0.80	0.0210–0.1270	0.90–0.94	NM	NM	NM	StressTech REOLOGICA Instruments AB	[61]
1.00–5.00	0.0500–28.0000	0.53–0.95	0.1–1000	23	NM	RS100 Haake	[94]
0.10–2.00	0.0120–1.3200	0.50–0.80	NM	NM	NM	NM	[68]
0.10–2.00	0.0050–0.0560	0.85–0.94	62–1007	22	CC	Haake model VT 180	[38]
0.25–0.50	0.0331–0.0834	0.66–0.81	0–1000	25–75	NM	Advanced Rheometric Expansion System (ARES)	[28]
0.25–2.00	0.0270–4.2200	0.56–0.84	0–2000	30	NM	RV-3 HAAKE	[33]
0.15–0.4	0.246–0.733	0.421–0.481	NM	20	NM	NM	[44]
0.10–0.25	0.0022–0.0069	0.85–0.95	NM	29	T	pipeline viscometer	[67]
0.05–0.10	0.0012–0.0023	0.81–0.84	NM	29	T	pipeline viscometer	[65]
0.01–0.20	0.0022–0.1690	0.63–0.95	163–3254	25	CC	model 35 Farm	[35]
0.03–0.50	0.0079–0.5940	0.56–0.84	163–3254	25	CC	model 35 Farm	[27]
0.10–0.60	0.0080–1.0050	0.52–0.90	100–4000	20–50	CP	PHYSICA model MCR30	[95]
0.2–0.4	0.054–0.209	0.73–0.805	NM	NM	CC	Fann Model 35	[39]
0.01–0.50	0.0036–0.6550	0.54–0.91	1.5–3254	50	CC	model 35 Farm	[29]
0.10–1.00	0.0121–4.3326	0.43–0.91	0–4000	29–25	NM	AR 2000 - TA Instruments	[62]
0.15–0.30	0.0110–0.0390	0.80–0.93	0.45–660	20	NM	NM	[70]
0.50–2.00	0.0660–7.0690	0.58–0.86	NM	20	T	capillary tube viscometer	[96]
0.10–0.30	0.0170–0.0800	0.77–0.85	NM	23	NM	Haake RV1	[97]
0.10–0.30	0.0890–0.9720	0.62–0.80	NM	20	DG	ThermoHaake RS300	[56]
0.05–0.35	0.0340–2.4340	0.54–0.95	NM	20	DG	ThermoHaake RS300	[58]
0.40	0.3720	0.73	NM	20	NM	NM	[13]
0.05–0.25	0.0025–0.0059	0.92–0.93	NM	25	NM	NM	[60]

CC: Concentric cylinder, CP: Cone-and-plate, DG: Double gap, NM: not mentioned, T: Tubular.

**Table 5:** Regression results for *k* vs. wt for the CMC dataset.

Set	Expression	RSE	$R_{\mathrm{adj}}^{2}$	${\mathrm{AIC}}_{c}$	${\mathrm{BIC}}_{c}$	*p*-Value
dSet	k∼wt	3.274	0.502	643.8192	649.4436	2.11 × 10^−20^
dSet	$k∼wt+k_{o}$	3.068	0.529	628.8310	637.2676	9.54 × 10^−22^
log(dSet)	k∼wt	1.543	0.784	458.6816	464.3059	1.39 × 10^−42^
log(dSet)	$k∼wt+k_{o}$	1.514	0.591	455.0516	463.4881	1.74 × 10^−25^
$dSet^{1/3}$	k∼wt	0.407	0.776	130.7712	136.3956	1.23 × 10^−41^
$dSet^{1/3}$	$k∼wt+k_{o}$	0.348	0.640	93.5095	101.9461	7.49 × 10^−29^
dSet	$k∼a\cdot wt^{b}$	1.995	0.733	522.9911	531.4277	9.32 × 10^−37^
$dSet^{1/3}$	$k∼a\cdot wt^{b}$	0.270	0.743	30.8134	39.2500	9.60 × 10^−38^
dSet	k∼a·exp(wt·b)	1.951	0.708	517.3987	525.8352	2.29 × 10^−34^
log(dSet)	k∼a·exp(wt·b)	0.462	0.264	163.2936	171.7302	7.57 × 10^−10^
$dSet^{1/3}$	k∼a·exp(wt·b)	0.248	0.765	10.2068	18.6433	4.03 × 10^−40^

*dSet*, dataset; *k*: Flow consistency index; *wt*: concentration (wt.%); *a*, *b*, *k_o_*: unknown parameters.

**Table 6:** Regression results for *k* vs. *T* for the CMC dataset.

Set	Expression	RSE	$R_{\mathrm{adj}}^{2}$	${\mathrm{AIC}}_{c}$	${\mathrm{BIC}}_{c}$	*p*-Value
dSet	k∼T	4.5420	0.0413	724.3454	729.9698	1.34 × 10^−2^
dSet	$k∼T+k_{o}$	4.4681	0.0017	721.2953	729.7318	2.73 × 10^−1^
log(dSet)	k∼T	2.3573	0.4947	563.0005	568.6249	5.09 × 10^−20^
log(dSet)	$k∼T+k_{o}$	2.3670	0.0010	565.0005	573.4371	2.91 × 10^−1^
$dSet^{1/3}$	k∼T	0.5938	0.5222	223.8186	229.4430	1.63 × 10^−21^
$dSet^{1/3}$	$k∼T+k_{o}$	0.5784	0.0072	218.3740	226.8106	1.73× 10^−1^
dSet	$k∼a\cdot T^{b}$	0.3919	0.0014	122.5989	131.0354	2.80 × 10^−1^
log(dSet)	$k∼a\cdot T^{b}$	0.2242	0.0010	−14.8137	−6.3772	2.91 × 10^−1^
$dSet^{1/3}$	$k∼a\cdot T^{b}$	0.0654	0.0057	−317.7006	−309.2640	1.96 × 10^−1^
dSet	k∼a·exp(T·b)	0.4262	0.0023	143.2357	151.6722	2.51 × 10^−1^
log(dSet)	k∼a·exp(T·b)	0.2201	0.0007	−19.3121	−10.8755	2.99 × 10^−1^
$dSet^{1/3}$	k∼a·exp(T·b)	0.0677	0.0065	−309.2259	−300.7893	1.83 × 10^−1^

*dSet*, dataset; *k*: Flow consistency index; *T*: Temperature; *a*, *b*, *k_o_*: unknown parameters.

**Table 7:** Best regression results for *k* as a function of $w_{t}$ and *T* for the CMC dataset.

Set	Expression	RSE	$R_{\mathrm{adj}}^{2}$	${\mathrm{AIC}}_{c}$	${\mathrm{BIC}}_{c}$	*p*-Value
$dSet^{1/3}$	k∼wt+T+wt·T	0.3515	0.8325	96.8232	108.0719	4.97 × 10^−47^
$dSet^{1/3}$	$k∼a\cdot wt^{b}+c\cdot T$	0.2486	0.7625	10.6013	19.0379	8.41 × 10^−40^

**Table 8:** Regression results for *n* vs. wt for the CMC dataset.

Set	Expression	RSE	$R_{\mathrm{adj}}^{2}$	${\mathrm{AIC}}_{c}$	${\mathrm{BIC}}_{c}$	*p*-Value
dSet	$n∼wt+n_{o}$	0.1322	0.2367	−144.705	−136.268	6.99 × 10^−9^
log(dSet)	$n∼wt+n_{o}$	0.1858	0.2885	−61.058	−52.621	9.04 × 10^−11^
$dSet^{1/3}$	$n∼wt+n_{o}$	0.0539	0.2983	−365.250	−356.814	3.87 × 10^−11^
dSet	$n∼a\cdot wt^{b}$	0.0667	0.2663	−313.134	−304.697	6.05 × 10^−10^
$dSet^{1/3}$	$n∼a\cdot wt^{b}$	0.0291	0.2811	−517.189	−508.752	1.71 × 10^−10^
dSet	n∼a·exp(wt·b)	0.0675	0.2475	−310.254	−301.817	2.88 × 10^−9^
log(dSet)	n∼a·exp(wt·b)	0.1030	0.3070	−206.011	−197.575	1.80 × 10^−11^
$dSet^{1/3}$	n∼a·exp(wt·b)	0.0297	0.2979	−511.997	−503.561	4.00 × 10^−11^

*dSet*, dataset; *n*: Flow behavior index; *wt*: Concentration (wt.%); *a*, *b*, *n_o_*: unknown parameters.

**Table 9:** Different rheological parameters for XG from the available literature.

Ref.	Concentration Range (wt.%)	*k* Range (*Pa s^n^*)	*n* Range (–)	Shear Rate Range (s^−1^)	*T* (°C)	Geometry	Rheometer
[41]	0.3–0.4	NM	0.2–0.45	0.001–10	23	CC	HR2 Discovery
[78]	0.3–0.6	0.6017–1.275	0.283–0.3712	0.001–100	20	CC	HR3
[81]	0.2	0.066	0.54	NM	NM	NM	NM
[92]	0.10–0.21	0.1110–0.3541	0.44–0.54	0–1000	16	DG	RHEOTEC RC 20
[76]	0.15	0.0075	0.9463	2.7–300	25	CC	Haake Viscotester 550
[72]	0.10–0.50	0.1000–2.9300	0.19–0.52	NM	16	NM	NM
[30]	0.01–0.20	0.0049–0.7076	0.32–0.84	NM	28	NM	Brookfield LVT -Synchro-electric
[98]	0.01–0.75	0.0015–546.7029	0.66–0.96	0–4000	20	CP	AR 1000N
[35]	0.01–0.20	0.0025–0.2050	0.49–0.91	163–3254	25	CC	model 35 Farm
[27]	0.10	0.0675	0.63	163–3254	25	CC	model 35 Farm
[95]	0.10–0.20	0.0790–0.3700	0.39–0.55	100–4000	20–50	CP	PHYSICA model MCR30
[29]	0.25–0.50	0.3740–2.1000	0.27–0.44	1.5–3254	50	CC	model 35 Farm
[59]	0.20	0.1685	0.52	NM	20	NM	NM

CC: Concentric cylinder, CP: Cone-and-plate, DG: Double gap, NM: not mentioned, T: Tubular

**Table 10:** Regression results for *k* vs. wt for the XG dataset.

Set	Expression	RSE	$R_{\mathrm{adj}}^{2}$	${\mathrm{AIC}}_{c}$	${\mathrm{BIC}}_{c}$	*p*-Value
log(dSet)	k∼wt	2.0101	0.6742	185.061	188.584	5.29 × 10^−12^
log(dSet)	$k∼wt+k_{o}$	1.2882	0.8109	147.761	153.045	1.23 × 10^−16^
$dSet^{1/3}$	k∼wt	1.3796	0.4536	152.693	156.215	3.28 × 10^−7^
$dSet^{1/3}$	$k∼wt+k_{o}$	1.1969	0.4514	141.439	146.723	4.88 × 10^−7^
dSet	$k∼a\cdot wt^{b}$	35.4541	0.8298	432.849	438.133	1.41 × 10^−17^
$dSet^{1/3}$	$k∼a\cdot wt^{b}$	0.7525	0.7351	101.531	106.814	1.30 × 10^−13^
log(dSet)	k∼aexp(wt·b)	0.8470	0.4524	111.699	116.982	4.69 × 10^−7^
$dSet^{1/3}$	k∼aexp(wt·b)	0.7238	0.7437	98.180	103.464	6.58 × 10^−14^

*dSet*, dataset; *k*: Flow consistency index; *wt*: Concentration (wt.%); *a*, *b*, *k_o_*: unknown parameters.

**Table 11:** Regression results for *k* as a function of wt and *T* for the XG dataset.

Set	Expression	RSE	$R_{\mathrm{adj}}^{2}$	${\mathrm{AIC}}_{c}$	${\mathrm{BIC}}_{c}$	*p*-Value
log(dSet)	k∼wt+T+w·T	1.363	0.8502	153.563	160.607	3.66 × 10^−17^
log(dSet)	$k∼wt+T+w\cdot T+k_{o}$	1.243	0.8240	146.543	155.349	2.14 × 10^−15^
$dSet^{1/3}$	$k∼a\cdot wt^{b}+c\cdot T$	0.720	0.7383	97.714	102.998	1.01 × 10^−13^
log(dSet)	$k∼a+b\cdot wt+c\cdot T+d\cdot wt^{2}+e\cdot T^{2}+f\cdot wt\;T$	0.833	0.9136	110.281	115.565	1.23 × 10^−23^
$dSet^{1/3}$	$k∼a+b\cdot wt+c\cdot T+d\cdot wt^{2}+e\cdot T^{2}+f\cdot wt\;T$	0.669	0.7826	91.385	96.669	2.20 × 10^−15^

*dSet*, dataset; *k*: Flow consistency index; *wt*: Concentration (wt.%); *T*: temperature; *a*, *b*, *c*, *d*, *e*, *f*, *k_o_*: unknown parameters.

**Table 12:** Regression results for *n* vs. wt for the XG dataset.

Set	Expression	RSE	$R_{\mathrm{adj}}^{2}$	${\mathrm{AIC}}_{c}$	${\mathrm{BIC}}_{c}$	*p*-Value
dSet	$n∼wt+n_{o}$	0.1974	0.12404	−13.5693	−8.286	1.18 × 10^−2^
log(dSet)	$n∼wt+n_{o}$	0.3364	0.35920	32.2762	37.560	1.30 × 10^−5^
$dSet^{1/3}$	$n∼wt+n_{o}$	0.0900	0.31885	−81.0937	−75.810	4.78 × 10^−5^
dSet	$n∼a\cdot wt^{b}$	0.1070	0.40168	−66.1891	−60.906	3.04 × 10^−6^
$dSet^{1/3}$	$n∼a\cdot wt^{b}$	0.0540	0.37371	−124.9583	−119.675	7.99 × 10^−6^
dSet	n∼aexp(wt·b)	0.1023	0.19075	−70.1112	−64.828	2.00 × 10^−3^
log(dSet)	n∼aexp(wt·b)	0.1814	0.31894	−20.8249	−15.541	4.76 × 10^−5^
$dSet^{1/3}$	n∼aexp(wt·b)	0.0538	0.33476	−125.3890	−120.105	2.88 × 10^−5^

*dSet*, dataset; *n*: Flow behavior index; *wt*: Concentration (wt.%); *a*, *b*, *n_o_*: unknown parameters

**Table 13:** Regression results for *n* as a function of wt and *T* for the XG dataset.

Set	Expression	RSE	$R_{\mathrm{adj}}^{2}$	${\mathrm{AIC}}_{c}$	${\mathrm{BIC}}_{c}$	*p*-Value
dSet	$n∼a\cdot wt^{b}+c\cdot T$	0.1074	0.4229	−65.927	−60.644	1.42 × 10^−6^
$dSet^{1/3}$	n∼aexp(wt·b)+c·T	0.0538	0.3417	−125.341	−120.057	2.31 × 10^−5^
dSet	$n∼a+b\cdot wt+c\cdot T+d\cdot wt^{2}+e\cdot T^{2}+f\cdot wt\;T$	0.1066	0.5161	−66.581	−61.297	3.51 × 10^−8^
log(dSet)	$n∼a+b\cdot wt+c\cdot T+d\cdot wt^{2}+e\cdot T^{2}+f\cdot wt\;T$	0.2107	0.5571	−7.956	−2.672	5.54 × 10^−9^
$dSet^{1/3}$	$n∼a+b\cdot wt+c\cdot T+d\cdot wt^{2}+e\cdot T^{2}+f\cdot wt\;T$	0.0545	0.5659	−124.170	−118.886	3.64 × 10^−9^

*dSet*, dataset; *k*: Flow consistency index; *wt*: Concentration (wt.%); *T*: temperature; *a*, *b*, *c*, *d*, *e*, *f*: unknown parameters.

**Table 14:** Different rheological parameters for AS available in the literature.

Ref.	Concentration Range (g/L)	*k* Range (*Pa s^n^*)	*n* Range (–)	Shear Rate Range (s^−1^)	*T* (°C)	Geometry	Rheometer
[73]	0.712–15.86	0.00528–0.05371	0.6887–0.4136	7.7–225.4	22	CC	Bohlin rheometer
[78]	2–3.6	0.64–6.788	0.278–0.35	0.001–100	20	CC	HR3
[6]	8	0.0524	0.59	0–1000	25	CC	Haake Rheostress RS 75
[69]	2.5–12.1	0.042–5.885	0.71–0.367	3–702	35	NM	NM
[7]	10	0.0130	0.72	10–1000	20	CC	Rotovisco RV 100 Haake
[21]	2.8–10.2	0.0037–0.1245	0.4385–0.8268	0.001–500	20	T	NM
[40]	3.23	0.296	0.606	1–1000	25	CC	SR2000
[11]	2.9–12.3	0.2783–3.0809	0.29–0.51	NM	15–30	NM	NDJ-Model
[12]	2.74–31	0.1710–5740	0.01–0.10	0–1000	15–25	NM	NM
[14]	3.7–22.9	0.0053–0.0629	0.64–0.89	3–1300	20	CC	Rheotest 2.1, Haaken Medingen GMBH
[18]	27–57	0.4873–13.2119	0.20–0.62	0–3000	20	CC & DG	AR 550
[16]	3–30	0.0133–0.1132	0.58–0.82	3–1300	20	CC	Rheotest 2.1, Haaken Medingen GMBH
[10]	2.7–47	0.0202–16.2516	0.04–0.67	0–2200	21	DG	VT 550 Haake
[39]	17.99	0.0036	0.073	NM	NM	CC	Fann Model 35
[8]	3.3–28.6	0.2842–1.1940	0.18–0.53	0–1850	25	NM	NM
[13]	2.74–16.00	0.0090–1.2590	0.25–0.73	25–1000	20	NM	AR 2000
[9]	6–16	0.0059–0.0068	0.56–0.91	NM	NM	NM	NM

CC: Concentric cylinder, CP: Cone-and-plate, DG: Double gap, NM: not mentioned, T: Tubular.

**Table 15:** Regression results for *k* vs. wt for the AS dataset.

Set	Expression	RSE	$R_{\mathrm{adj}}^{2}$	${\mathrm{AIC}}_{c}$	${\mathrm{BIC}}_{c}$	*p*-Value
dSet	k∼wt	2.3341	0.615	660.302	666.255	7.05 × 10^−32^
dSet	$k∼wt+k_{o}$	2.1518	0.574	637.707	646.637	1.59 × 10^−28^
log(dSet)	$k∼wt+k_{o}$	1.8867	0.290	599.571	608.502	1.67 × 10^−12^
$dSet^{1/3}$	$k∼wt+k_{o}$	1.7555	0.096	590.572	599.564	7.80 × 10^−5^
dSet	$k∼a\cdot wt^{b}$	1.5170	0.714	536.322	545.252	6.13 × 10^−41^
$dSet^{1/3}$	$k∼a\cdot wt^{b}$	0.3398	0.515	102.437	111.367	1.86 × 10^−24^
dSet	k∼aexp(wt·b)	1.4518	0.680	523.595	532.525	2.16 × 10^−37^
log(dSet)	k∼aexp(wt·b)	0.5100	0.288	220.181	229.111	2.17 × 10^−12^
$dSet^{1/3}$	k∼aexp(wt·b)	0.3232	0.556	87.933	96.864	3.15 × 10^−27^

*dSet*, dataset; *k*: Flow consistency index; *wt*: Concentration (wt.%); *a*, *b*, *k_o_*: unknown parameters.

**Table 16:** Regression results for *k* as a function of wt and *T* for the AS dataset.

Set	Expression	RSE	$R_{\mathrm{adj}}^{2}$	${\mathrm{AIC}}_{c}$	${\mathrm{BIC}}_{c}$	*p*-Value
dSet	k∼wt+T+wt·T	2.1505	0.6733	638.512	650.419	5.68 × 10^−35^
$dSet^{1/3}$	k∼wt+T+wt·T	0.4553	0.8168	188.300	200.207	9.11 × 10^−53^
dSet	$k∼wt+T+wt\cdot T+k_{o}$	2.1578	0.5720	640.469	655.353	1.73 × 10^−26^
$dSet^{1/3}$	$k∼wt+T+wt\cdot T+k_{o}$	0.4564	0.4327	189.940	204.823	6.39 × 10^−18^
dSet	$k∼a\cdot wt^{b}+c\cdot T$	1.4870	0.7149	530.536	539.466	5.10 × 10^−41^
$dSet^{1/3}$	$k∼a\cdot wt^{b}+c\cdot T$	0.2842	0.2703	50.685	59.615	1.24 × 10^−11^
dSet	$k∼a+b\cdot wt+c\cdot T+d\cdot wt^{2}+e\cdot T^{2}+f\cdot wt.T$	1.4088	0.7607	514.869	523.799	1.84 × 10^−46^
$dSet^{1/3}$	$k∼a+b\cdot wt+c\cdot T+d\cdot wt^{2}+e\cdot T^{2}+f\cdot wt.T$	0.2914	0.6398	57.929	66.859	9.89 × 10^−34^

*dSet*, dataset; *k*: Flow consistency index; *wt*: Concentration (wt.%); *T*: temperature; *a*, *b*, *c*, *d*, *e*, *f*: unknown parameters.

**Table 17:** Regression results for *n* as a function of wt and *T* for the AS dataset.

Set	Expression	RSE	$R_{\mathrm{adj}}^{2}$	${\mathrm{AIC}}_{c}$	${\mathrm{BIC}}_{c}$	*p*-Value
dSet	$n∼a\cdot wt^{b}+c\cdot T$	0.0787	0.1467	−321.760	−312.830	1.19 × 10^−6^
log(dSet)	n∼aexp(wtcotb)+c·T	0.2447	0.0975	7.248	16.178	7.76 × 10^−5^
$dSet^{1/3}$	n∼aexp(wtcotb)+c·T	0.0614	0.1956	−393.840	−384.910	1.53 × 10^−8^
dSet	$n∼a+b\cdot wt+c\cdot T+d\cdot wt^{2}+e\cdot T^{2}+f\cdot wt.T$	0.1047	0.2743	−239.043	−230.113	8.35 × 10^−12^
log(dSet)	$n∼a+b\cdot wt+c\cdot T+d\cdot wt^{2}+e\cdot T^{2}+f\cdot wt.T$	0.3343	0.2712	97.712	106.642	1.13 × 10^−11^
$dSet^{1/3}$	$n∼a+b\cdot wt+c\cdot T+d\cdot wt^{2}+e\cdot T^{2}+f\cdot wt.T$	0.0725	0.3034	−345.688	−336.758	4.27 × 10^−13^

*dSet*, dataset; *k*: Flow consistency index; *wt*: Concentration (wt.%); *T*: temperature; *a*, *b*, *c*, *d*, *e*, *f*: unknown parameters.

## Data Availability

The data presented in this study are available on request from the corresponding author.

## Abbreviations

The following abbreviations are used in this manuscript:

AS	Activated Sludge
CAS	Conventional Activated Sludge
CFD	Computational Fluid Dynamics
CMC	Carboxymethyl Cellulose
DG-CC	Double Gap Concentric Cylinder geometry
ERT	Electrical Resistance Tomography
MBR	Membrane Bioreactors
MLSS	Mixed Liquor Suspended Solids
PEPT	Positron Emission Particle Tracking
PIV	Particle Imaging Velocimetry
PSD	Particle Size Distribution
PST	Pulse Shadow Technique
TSS	Total Suspended Solids
WWT	Wastewater treatment
XG	Xanthan Gum
